# Membrane anchoring facilitates colocalization of enzymes in plant cytochrome P450 redox systems

**DOI:** 10.1038/s42003-021-02604-1

**Published:** 2021-09-09

**Authors:** Tomas Laursen, Hiu Yue Monatrice Lam, Kasper Kildegaard Sørensen, Pengfei Tian, Cecilie Cetti Hansen, Jay T. Groves, Knud Jørgen Jensen, Sune M. Christensen

**Affiliations:** 1 Department of Plant and Environmental Sciences, University of Copenhagen, Copenhagen, Denmark; 2 Department of Chemistry, University of California, Berkeley, CA USA; 3 Department of Chemistry, University of Copenhagen, Copenhagen, Denmark; 4 Present Address: Enzyme Research, Lyngby, Denmark

**Keywords:** Membranes, Biophysical chemistry, Single-molecule biophysics

## Abstract

Plant metabolism depends on cascade reactions mediated by dynamic enzyme assemblies known as metabolons. In this context, the cytochrome P450 (P450) superfamily catalyze key reactions underpinning the unique diversity of bioactive compounds. In contrast to their soluble bacterial counterparts, eukaryotic P450s are anchored to the endoplasmic reticulum membrane and serve as metabolon nucleation sites. Hence, membrane anchoring appears to play a pivotal role in the evolution of complex biosynthetic pathways. Here, a model membrane assay enabled characterization of membrane anchor dynamics by single molecule microscopy. As a model system, we reconstituted the membrane anchor of cytochrome P450 oxidoreductase (POR), the ubiquitous electron donor to all microsomal P450s. The transmembrane segment in the membrane anchor of POR is relatively conserved, corroborating its functional importance. We observe dynamic colocalization of the POR anchors in our assay suggesting that membrane anchoring might promote intermolecular interactions and in this way impact assembly of metabolic multienzyme complexes.

## Abstract (web summary)

Laursen et al. investigates the role of membrane anchoring for the organization and efficiency of P450-driven plant metabolism. They report an original experimental design to monitor interactions between anchoring segments of membrane-bound enzymes at the single molecule level, using the cytochrome P450 oxidoreductase (POR) transmembrane segment as a model, and propose that membrane anchoring has played a key role in the evolution of P450- based metabolic pathways in plants.

## Introduction

Enzymes of the cytochrome P450 superfamily are involved in the majority of plant specialized metabolic pathways^1^. Almost all eukaryotic P450s are class II membrane proteins that are anchored to the endoplasmic reticulum via an N-terminal single transmembrane alpha helix and they depend on reducing power from a membrane-anchored NADPH-dependent cytochrome P450 oxidoreductase (POR)^2–4^. POR serves as a metabolic hub that activates specific P450 enzymes in response to chemical cues and local environment changes such as lipid composition and ionic strength^5–7^. In plants, typically 2-4 POR isoforms^8^ mediate electron transfer from NADPH to several hundred different P450 enzymes^9^. Furthermore, based on absolute quantities POR is stoichiometrically outnumbered to P450 enzymes with a factor of 5-20^10,11^, which points to dynamic interactions between POR and specific P450 isoforms

It is known that POR-P450 complexes are stabilized by electrostatic interactions between the globular domains^12,13^. However, the accretion of the anchor segment suggests a functional role in the organization of P450s and POR-mediated metabolism. The anchor may be directly involved in enzyme oligomerization as demonstrated by the dimerization of the transmembrane anchors of a mammalian P450 enzyme and a cytochrome b5 redox protein^14^. Additionally, membrane anchoring confines diffusion of the enzymes to the 2-dimensional membrane plane rather than the 3-dimensional cytosolic space, which may drive enzyme interactions^15–17^. Hence, the question remains: Is membrane anchoring directly linked to organization of P450 based metabolic cascades in plants, and thereby critical to the emergence of the chemical diversity we observe in the plant kingdom?

Here, we introduce a platform for studying the properties of isolated membrane anchors, thus providing insight into their putative role in the organization of enzyme complexes. We directly observe the diffusion and colocalization of chemically synthesized hydrophobic anchor peptides from POR in biomimetic lipid membranes by single molecule microscopy and particle tracking. As opposed to previous reconstitution strategies employing full-length proteins^18,19^ we chose this reductionistic approach to the problem in order to separate the effect of the anchor from interactions mediated by the globular domains. Interestingly, we find dynamic colocalization of the POR anchors at the membrane at physiological densities. Comparing empirical data with simulations we propose that membrane anchoring serves to enhance the colocalization of enzymes at the membrane and thereby promote intermolecular interactions. This might have been a key requirement for the evolution of multienzyme complexes involved in the biosynthesis of structurally advanced molecules.

## Results

### Reconstitution of mobile POR anchors in a model membrane system

**Fig. 1:**
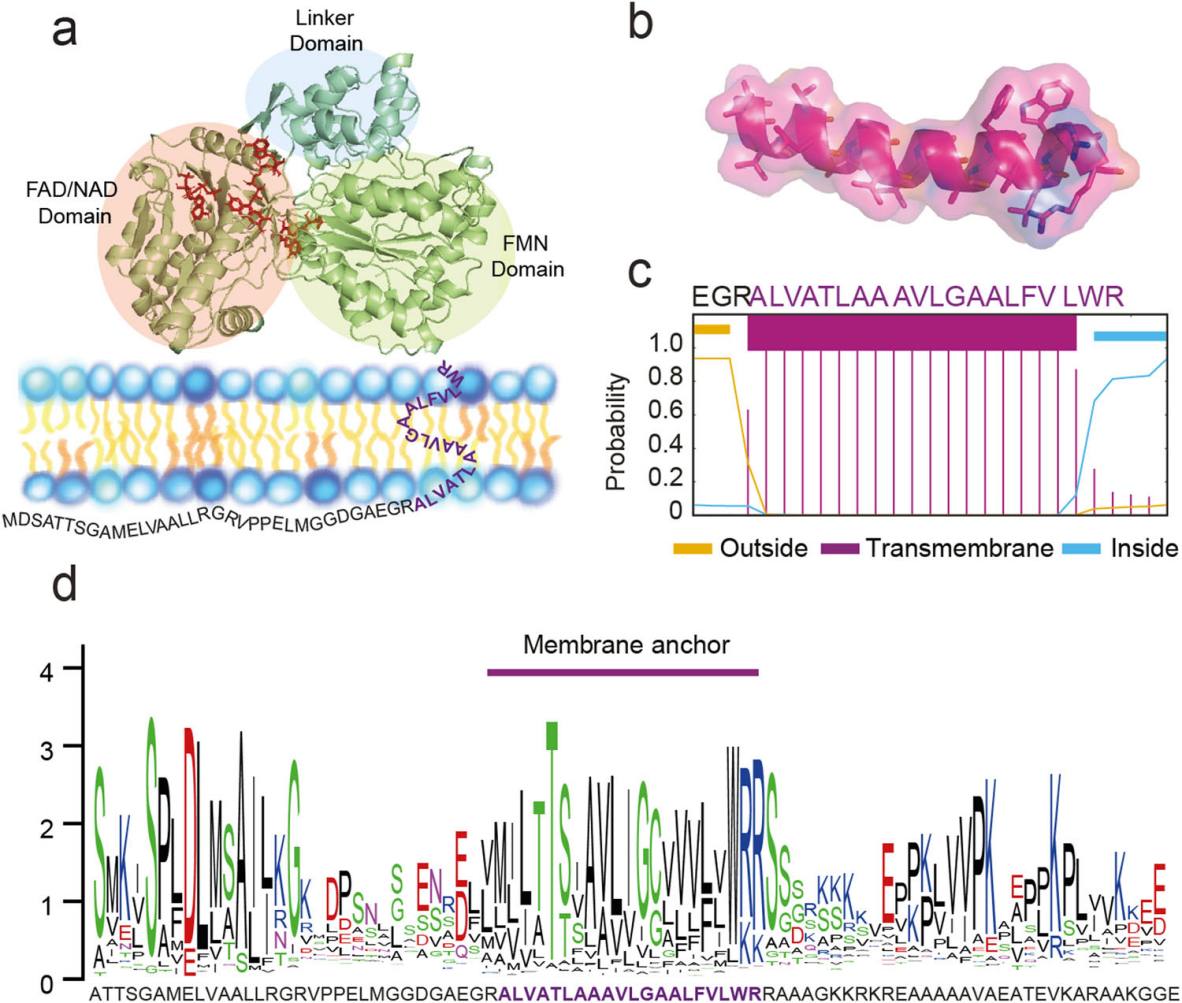
Membrane-bound Cytochrome P450 Oxidoreductase (POR). **a** Homology model of POR2b from *Sorghum bicolor* was performed using the automated online Phyre2 server. The three major domains are highlighted namely FMN binding domain, linker domain, and FAD/NADPH binding domain. The membrane anchor is depicted as the amino acid single letter code. **b** Structural model of the anchor region predicted by RaptorX, which is a protein structure prediction method based on homology modeling and deep learning. **c** Transmembrane α-helix prediction for the anchor region of POR2B by TMHMM, powered by a hidden Markov model^66^, with N-terminus (EGR) and C-terminus (RAAA). Source data available as Supplementary Data 3. **d** WebLogo representation of the conservation of POR sequences depicted from multiple sequence alignment of 278 homologous (Supplementary Fig. 1,^67^). The anchor region consensus sequence is MILTTSIAVLIGCVVVLVWR. The difference between the maximum possible entropy and the entropy of the observed amino acid distribution in the anchor region is higher than for the adjacent linker regions, indicating a higher degree of sequence conservation in this part of the protein. Data input for generating the WebLogo is available in Supplementary Data 4.

POR is a diflavin enzyme binding one molecule each of flavin mononucleotide (FMN) and flavin adenine dinucleotide (FAD) with a highly conserved tertiary structure composed of an FMN domain, a linker domain interconnected with a flexible loop, and NADPH and FAD binding domain^20^ (Fig. 1a). It has likely evolved from a fusion of two bacterial ancestral soluble proteins, flavodoxin and ferredoxin NADP-oxidoreductase^21,22^. Furthermore, eukaryotic PORs are tethered to the endoplasmic reticulum via an N-terminal single transmembrane anchor (Fig. 1b and c). The structural organization and amino acid sequence of POR remains highly conserved among the globular domains, i.e., FMN-, Linker- and FAD/NADPH-domains (Supplementary Fig. 1). In contrast, the N-terminal part shows higher variability but, interestingly, the transmembrane segment of the POR anchor shows a relatively high degree of conservation across plant species (Fig. 1d) thus indicating evolutionary pressure and functional importance.

We have established a model membrane system for direct single molecule observation of reconstituted POR membrane anchor dynamics. A 36 amino acid peptide comprising the transmembrane segment of *Sorghum bicolor* POR2B was synthesized with limited amino acids protruding on each side of the membrane and with an amino terminal cysteine residue amenable for fluorescence labeling. The peptide was assembled by solid-phase peptide synthesis with a C terminal amide. The presence of the C-terminal amide avoids the introduction of a negative charge at the C-terminal, which might reduce the cell-penetrating property of the peptide. The N-terminal was acetylated to prevent the formation of byproducts during attachment of fluorophores and to ease purification of the peptides. Purification was done by preparative HPLC (Supplementary Fig. 2) prior to attachment of ATTO dyes via alkylation at the cysteine side-chain. The peptide was labeled with either ATTO488 (POR-488) or ATTO647N (POR-647N) (Supplementary Figs. 3, 4). Except where otherwise noted, presented data pertain to the ATTO647N labeled peptide, which exhibited superior photostability and thus yielded the best data quality. The ATTO488 was included to investigate labeling effects and gauge the potential in future dual-color multi-peptide diffusion experiments. The POR anchors were reconstituted in vesicles at biologically relevant density of 31/μm^2^ (see Supplementary Note 1) by mixing the peptide with phospholipids in chloroform followed by rehydration.

**Fig. 2:**
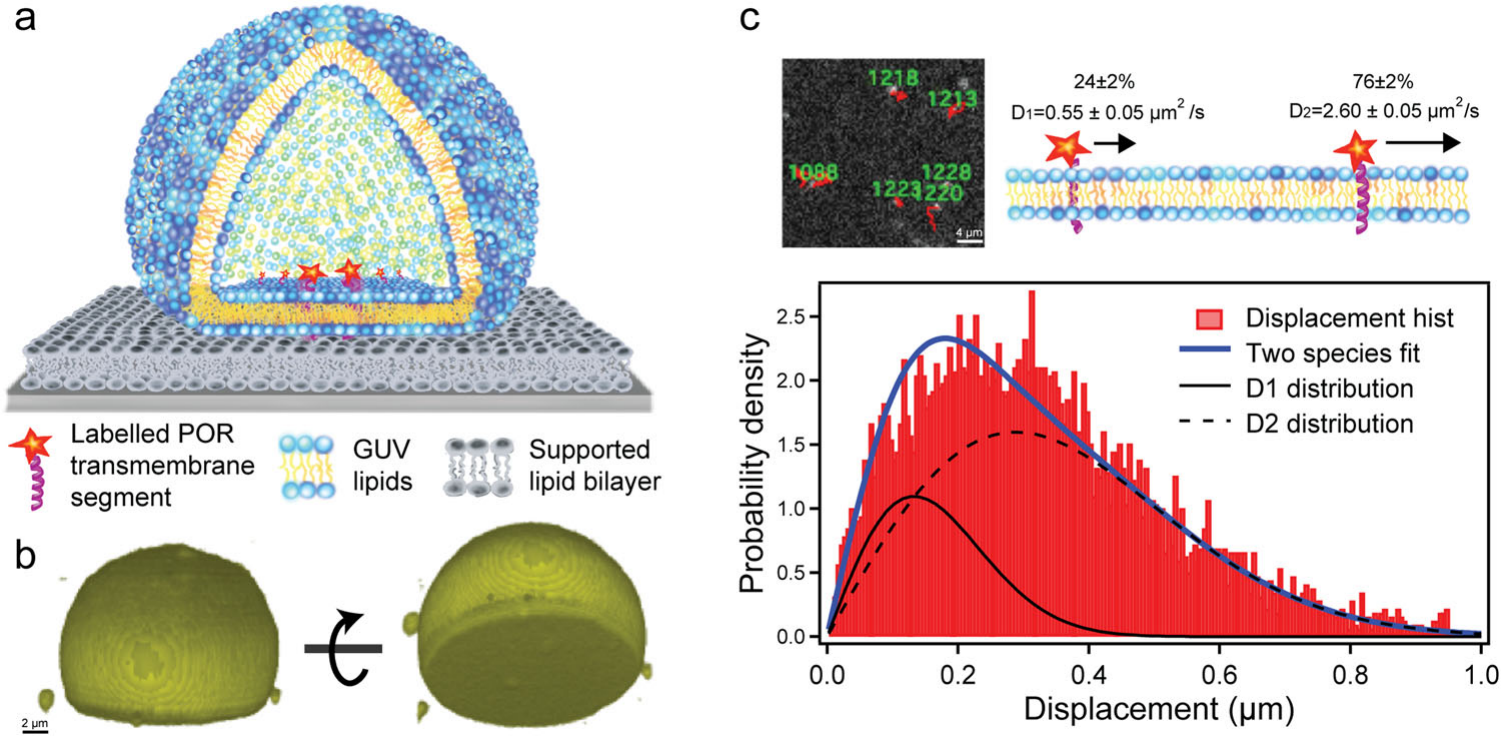
Reconstitution of POR transmembrane anchor segment in a fluid lipid membrane system. **a** Synthesized fluorescently labeled anchors are reconstituted in Giant Unilamellar Vesicles (GUVs) gravitationally settled on a supported lipid bilayer. **b** Three-dimensional reconstruction of a fluorescently labeled GUV based on confocal microscopy. **c** Single molecule tracking by Total Internal Reflection Fluorescence (TIRF) microscopy of anchors diffusing in GUV membrane interfaced with a supported bilayer. Single molecule displacement data were fitted with a free Brownian diffusion model (blue trace on graph) yielding two diffusing species with diffusion coefficients and weights as indicated. The errors indicate the 95% Highest Density Interval (HDI) of the posterior probability distribution for the diffusion coefficients (fits were based on 11,444 displacement, time pairs collected from tracking experiments with *n* = 3 different GUVs from two independent samples. The histogram show displacements for a fixed 16 ms time lapse (6,355 data points) - see Supplementary Table 1 for additional information including POR-488 data). Source data are available in Supplementary Data 5.

Initial attempts to reconstitute POR anchors in supported lipid bilayers resulted in immobilization, likely due to non-specific interactions with the glass. It is not uncommon that transmembrane peptides are prone to immobilization in supported bilayers^23,24^. Strategies to overcome this include cushioned bilayers^25^ and formation of multi-layered structures^26^. Here we circumvented the issue using stacked lipid bilayers^27^ created by gravitational settling of Giant Unilamellar Vesicles (GUVs) on a glass slide passivated with a supported bilayer, Fig. 2a, b. In this way, mobile POR anchors were readily reconstituted by inclusion in the GUV membrane.

### Single molecule tracking reveals two mobility modes

The GUVs formed a dome with the planar surface facing the support enabling single molecule imaging by Total Internal Reflection Fluorescence (TIRF) microscopy that confines fluorophore excitation to the first few hundred nm above the glass surface. This allowed us to directly observe the movements of individual anchors and characterize their mobility, Fig. 2c. Interframe displacements were collected for all single molecule trajectories in the analyzed time series and fitted with a free Brownian diffusion model using a previously published maximum likelihood procedure (^28^ and Supplementary Note 2). We found that two characteristic Brownian diffusion modes were needed to adequately describe the data, which was substantiated by model selection based on the Bayesian Information Criterion (BIC) (^29^ and Supplementary Table 1). As a control, data from stochastic simulations with a single diffusion coefficient yielded a single diffusion mode when subjected to the same analytical framework (Supplementary Table 1).

For POR-647N we found D_1 _= 0.55 ± 0.05 µm^2^/s and D_2 _= 2.60 ± 0.05 µm^2^/s with the fraction *α* = 24 ± 2% of data pertaining to the D_1_ distribution. POR-488 exhibited slightly slower mobility with D_1 _= 0.10 ± 0.05 µm^2^/s, D_2 _= 1.15 ± 0.08 µm^2^/s and *α* = 11 ± 2% (errors comprise the 95% Highest Density Interval (HDI) of the posterior probability distribution used for parameter estimation, see Supplementary Fig. 5 and Supplementary Table 1). While the fast observed diffusion species is in the range of expected mobility in GUVs^30^ the second species is in both cases considerably slower. The physical nature of the two diffusion modes cannot be inferred from the displacement analysis but similar observations for proteins in supported membranes have previously been linked to oligomerization^19,28^.

### Colocalization of POR anchors at the membrane

**Fig. 3:**
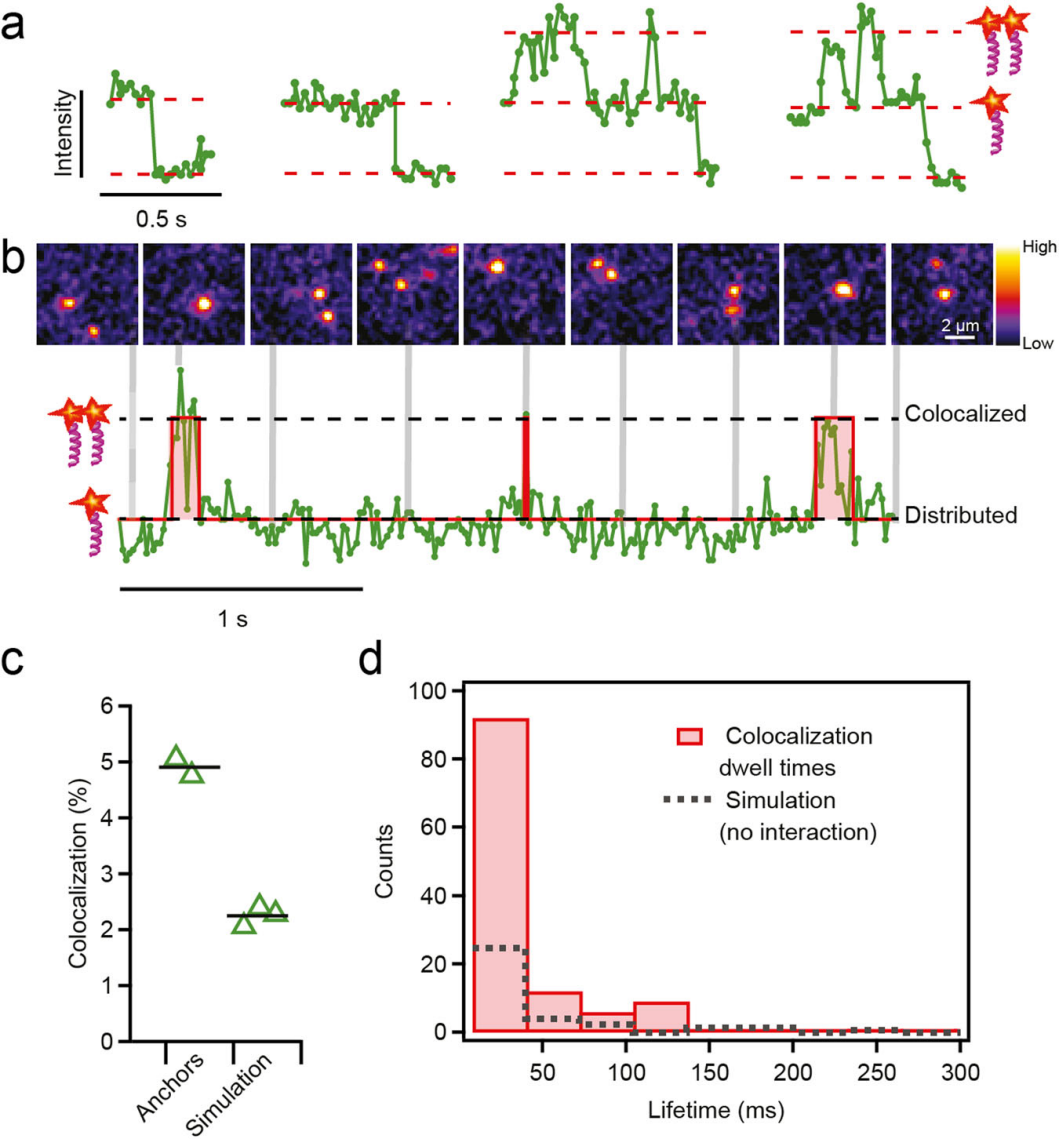
Colocalization of POR anchors at the membrane. **a** Representative single molecule intensity traces. The first two traces exhibit single-step photobleaching whereas the last two traces show regions with double brightness, indicative of dynamic colocalization. **b** An extended single molecule intensity trace and corresponding micrograph snapshots with timings indicated by the vertical grey lines. Regions of double brightness in the intensity trace corresponds to colocalization of two anchors in the micrographs. The intensity trace is segmented and fitted with a two state model (red trace) employing a change point detection algorithm (see methods). To ease visualization shown micrographs were smoothed using a 3×3 Gaussian kernel. **c** Colocalization of reconstituted anchors compared to coincident colocalization due to random overlap. Each triangle reflects colocalization percentage calculated from trajectories from a single movie/GUV, or a single simulation run, and employing the analysis procedure illustrated in (**b**). **d** Histograms of colocalization event durations. Single molecule trajectories from the two GUVs included in panel c were joined and analyzed to extract colocalization event dwell times (121 events total). The average histogram of 3 corresponding traces resulting from the simulated data is shown for comparison, each with the same length as the original data being compared here (average number of events per simulated trace was 34 with individual traces showing 22, 34, and 47 events). Source data are available in Supplementary Data 6.

The brightness dimension of single molecule video streams can be exploited as a direct reporter of protein colocation on membranes^28,31^. We integrated fluorescence intensity along the trajectories using a sliding Region Of Interest (ROI) of 5 × 5 pixels (790 × 790 nm) generating traces of the form Fig. 3a. Most traces exhibited characteristic single step photobleaching profiles, as shown for the first two examples in Fig. 3a, but some traces were found to explore two evenly spaced brightness levels. Joint inspection of brightness and image data revealed that the second observed plateau indeed corresponds to two molecules coming together, co-diffusing and eventually splitting paths, Fig. 3b (see also Supplementary Fig. 6 for additional data for POR-488).

We quantified the observed colocalization by segmenting traces into distributed and colocalized regions using a change point detection algorithm^32,33^. the analysis shows that molecules were together on average 4.9% of the observation time, Fig. 3c. Figure 3d provides a histogram of dwell times for the identified colocalized states. Colocalization naturally comprise contributions from coincident random overlaps and potential physical molecular interactions. We determined the relative contribution of each by generating tracking movies based on stochastic simulations with input parameters given by the original data (diffusivity, particle speckle width, amplitude, and density – see Methods). Applying the same tracking and analysis procedure used for the original video streams to the simulated data yielded an average of 2.25% colocalization, Fig. 3c. We conclude that the POR anchors come into proximity at the membrane both as a statistical consequence of random motion and by physical means.

### Membrane grafting as a driver of intermolecular interactions in plant P450-based metabolism

Membrane localization is a hallmark of plant P450 based metabolic cascades, but in simpler organisms such as bacteria these reactions are cytosolic. The ability to multiplex and dynamically assemble enzymes in a combinatorial manner at the membrane has enabled a major advancement in terms of metabolic capability. But why would the physical constraint imposed by the membrane favor such an evolutionary arc? In signal transduction, it is well established that membrane recruitment can be a potent driver of enzyme interactions, with up to 1000-fold effective concentration enhancement depending on the conditions^15–17^. Inspired by our observation of dynamic colocalization of the POR anchors we hypothesize that a similar effect could be important in metabolon formation.

**Fig. 4:**
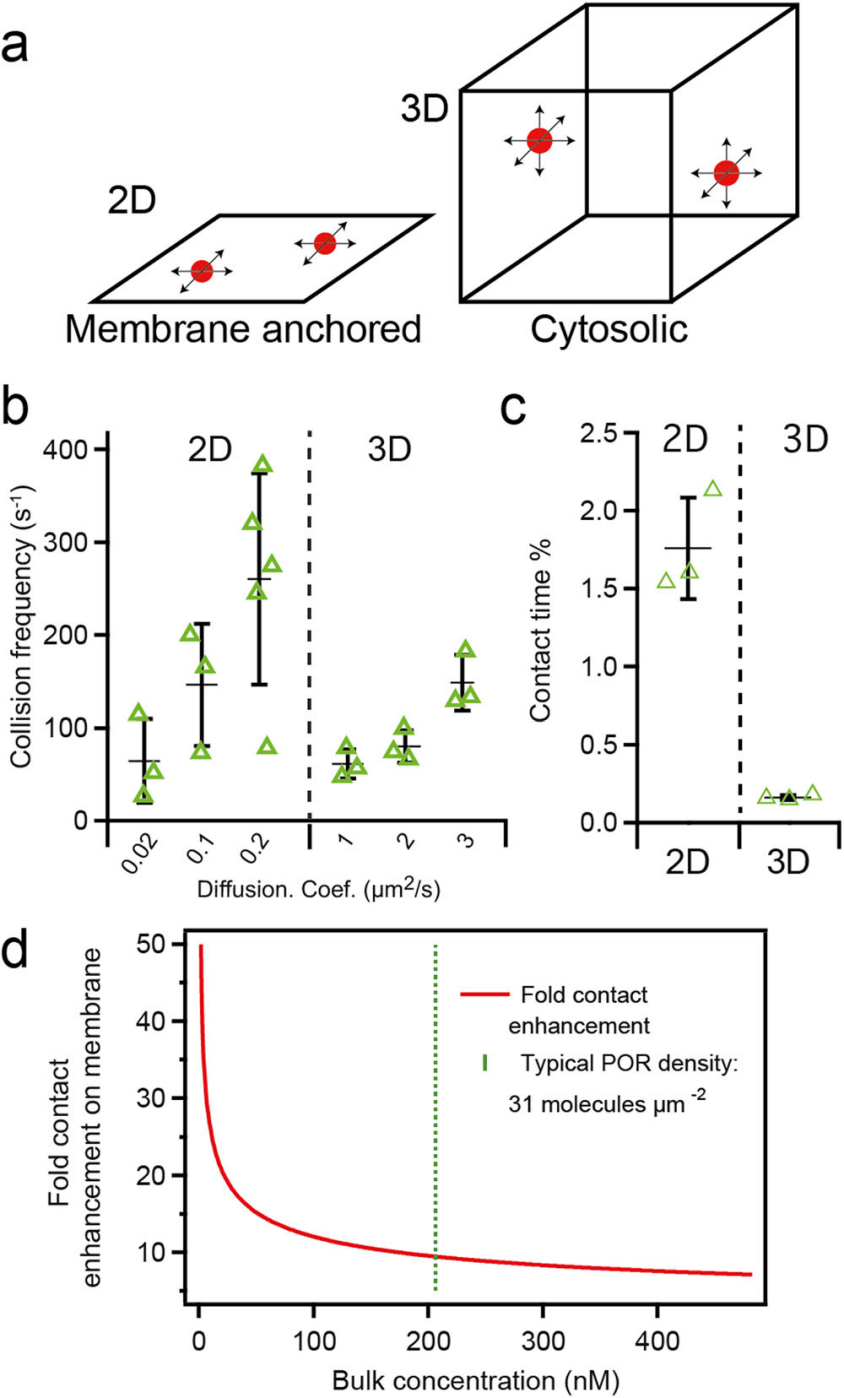
Membrane anchoring drives intermolecular interactions. **a** Stochastic diffusion simulation system with two particles confined to two- or three dimensions. In both cases, the side of the simulation box is *L* *=* 0.25 µm (corresponding to a membrane density of 31 molecules/µm^2^ and 206 nM, respectively), the box harbor two spherical particles with radius *R* *=* 10 nm. Time increments in the stochastic simulation were 10 µs and each simulation run was 750 ms. The two molecules were counted as being in contact when the center-to-center distance was ≤20 nm which is approximately the dimension of a putative POR dimer^20,34^. **b** Collision frequency for an array of typical diffusion coefficients comparing two- and three-dimensional diffusion. Each bar provides the average ± s.d. of *n* = 3 simulation runs, except *n* = 5 for *D* = 0.2 µm^2^/s. **c** Contact time of the two particles in two- and three dimensions (reported as % of time in contact relative to the full simulation time). Bars provide the average ± s.d of colocalization calculated for the 3 diffusion coefficient bins in (**b**) for the respective dimensionality. **d** Contact enhancement upon membrane grafting as a function of particle concentration in bulk. See Supplementary Note 3. Source Data are available in Supplementary Data 7.

We contextualize this discussion using a simple stochastic simulation framework. Two proteins were allowed to diffuse inside a cube with side *L* = 0.25 µm. In one case, the proteins were free to diffuse in the three dimensions of the cube (corresponding to a cytosolic concentration of 206 nM). In another situation, the proteins were constrained to move in the plane of one side of the cube (corresponding to a membrane density of 31 molecules µm^−2^), thus representing the cases of Brownian motion, respectively, in the cytosol and at a membrane at physiologically relevant density for POR (Fig. 4a, see also Supplementary Note 1 for a discussion of physiological densities). From the simulation we measured the collision frequency of the two proteins and the contact time percentage, that is the fraction of simulation time spend in physical contact. Physical contact was defined as the two proteins having a center-to-center distance of ≤20 nm, based on the geometric size of the globular domain of POR^20,34^.

We conducted the simulations with a range of typically reported diffusion coefficients for proteins in membranes (D_mem_ = 0.02, 0.1, 0.2 µm^2^/s) and in the cytosol (D_cyt_ = 1,2,3 µm^2^/s)^31,35^. The results show that membrane attachment, in general, had a minor impact, if any, on the collision frequency as the slower diffusion basically counteracts the increased hit probability in two dimensions (Fig. 4b). However, a major effect manifests as increased contact time when proteins are membrane-bound (ca. 10x for the particular case) (Fig. 4c). This effect arises because protein molecules bound to membranes spend a markedly higher fraction of their time in contact due to the reduced dimensionality but not because of an increased collision frequency. The membrane thus serves to enhance the effective protein concentration which can be a strong driver of intramolecular interactions, in analogy with findings from the signal transduction field^16^. The concentration enhancement is a purely geometrical effect and for our simple system, it is given analytically as, fold enhancement = 3 L/8 R (Supplementary Note 3), where L is the side of the cube and R the radius of the considered protein. The relation is shown as a function of cytosolic concentration in Fig. 4d.

## Discussion

Here, we introduce a platform for dissecting the role of the POR transmembrane anchor segment on colocalization and dynamic assembly by single molecule microscopy in a biomimetic membrane. The method relies on the chemical synthesis of the hydrophobic transmembrane anchors. Synthetic anchors were dissolved in lipids and reconstituted into GUVs, which were deposited by gravitation directly on a supported lipid bilayer serving as a cushion enabling free diffusion of the anchors (Fig. 2). GUVs are efficiently being adopted as biomimetic cell systems^27,36,37^ and the assay could potentially be generalized to studying alternative anchors or the trans interaction of different metabolic protein anchors and associated processes that might be important to cascade assembly.

Membrane anchors reconstituted in the GUVs were visualized by single molecule microscopy and tracking to reveal the diffusion by step size displacement (Fig. 2) and dynamic colocalization of anchors by brightness analysis (Fig. 3). The brightness analysis provides direct observation of molecular level colocalization and suggests that 2.65% of the trajectories reflect colocalization beyond what would be expected from purely statistical considerations. This observation might reflect direct physical interactions in the form of, e.g., dimerization, or alternative forms of physical co-partitioning within the membrane system^38^. However, the distribution is biased toward monomeric species due to pre-bleaching and the brightness-based colocalization is thus inevitably a conservative estimate of the degree of colocalization. Pre-bleaching is not an issue in our displacement-based classification of slow- and fast-moving populations because in this case, the “dark” population contribution is measurable as it still impacts the observed mobility for the unbleached minority. With the assumption that the slow diffusing species reflect the same underlying phenomenology driving the brightness-based colocalization a more liberal estimate for the proportion of colocated species would be in the range 11–24%, as observed for POR-488 and POR-647N anchors, respectively. A qualitative indication for a coupling of the two observations is provided by displacement analysis of data split based on the brightness-based trace segmentation (see Supplementary Fig. 7 and Supplementary Table 2). In the present study, reconstitution of anchors in GUVs renders a random orientation, which may affect the degree of colocalization. Regardless of the absolute proportion, the fundamental finding that the transmembrane anchor of POR is prone to colocalization in membranes at physiological densities corroborates the hypothesis that the POR anchor has evolved to serve a role in the spatial organization of the enzyme. Our experimental system opens an avenue toward quantitative assessment of how membrane anchors contribute to the organization of P450-based metabolism at membranes.

The hydrophobic transmembrane anchor segment is characteristic of both POR and the microsomal P450s. However, this segment is often neglected and readily exchanged for optimized expression in heterologous systems^39^ and omitted in structural analysis due to technical challenges^20,40,41^.

A comparison of 278 POR sequences from across land plant phylogeny showed that the anchor segment of POR remains conserved compared to the adjacent more flexible regions with a consensus sequence of N- MILTTSIAVLIGCVVVLVWR-C (Fig. 1d, Supplementary Fig. 1). Conservation of the anchor sequence strongly suggests that sequence identity plays a critical role. The membrane anchor is flanked by adjacent more unstructured flexible regions that display the canonical negatively charged residues like aspartate and glutamate on the N-terminal side and positively charged residues like arginine and lysine on the C-terminal side^42^. These charged residues are involved in the orientation of the anchor rendering the C-terminal globular domain facing the cytosol^43^. A proline rich segment on the C-terminal side of the anchor (Fig. 1d) may be involved in folding of the globular domain as demonstrated for the P450 CYP2C2^44^. The position of the membrane anchor relative to the globular domain is not well established due to lack of structural insight of full-length POR enzymes including the hydrophobic membrane anchor segment. A simulation study of a human P450 (CYP3A4) revealed the anchor to be positioned at the margin available for anchor-anchor interactions^45^. Thus, an intriguing regulatory level emerges, where the spatial organization of the enzyme(s) at the membrane could be orchestrated by the transmembrane anchoring motifs likely acting in concert with the enzyme’s globular domains.

In addition to the potential functional role of the anchor in protein–protein interactions, membrane anchoring is intimately linked to the concept of local concentration enhancement imposed by the mere geometrical constraints imposed by grafting proteins to cellular membranes^16,17,46^ (Fig. 4). We find that membrane anchoring of POR2B at physiological concentrations of ca. 30 molecules/μm^2^, corresponding to approximately 200 nM in solution, leads to ca. 10 fold effective concentration enhancement (Fig. 4d). This effect increases asymptotically at lower densities corresponding to the physiological concentration of the different P450 enzymes where an effect on the order of 50-, or higher, fold enhancement is expected based on our estimation (Fig. 4d). This is in agreement with the well-established general concept of local concentration enhancement that dictates that membrane anchoring of proteins present in the nM range at the membrane surface may increase their local effective concentration to the high µM range^46^. Exploiting this phenomenon might be key to the ability of plants to harness a diverse arsenal of P450s^9^ for metabolism with low expression to optimize resources. If the POR and P450 enzymes had not become membrane-anchored, as is the case for their bacterial counterparts, the ability to maintain a similar metabolic arsenal would require an up to 1,000 fold increase in expression level, which inevitably would become a significant resource burden for the cells.

Membrane anchoring may have been a requirement for the evolution of multienzyme pathways for the biosynthesis of bioactive compounds in higher organisms. Indeed, there is no clear evidence for the oligomerization of POR. However, in plants, POR may support electrons for metabolic pathways comprising multiple P450 enzymes, which could benefit from stoichiometric amounts of POR. These pathways convert simple general metabolites by sequential action of multiple enzymes, in some cases more than 30 enzymatic steps as demonstrated for the alkaloid vinblastine^47^, into an astonishing array of bioactive chemical compounds^48,49^. Organization of sequential enzymes into complexes termed metabolons facilitate substrate channeling and prevent leakage of potentially toxic and labile intermediates^50–52^. Although only a few pathways have been proven to organize as metabolons, assembly of sequential enzymes is thought to play a key role in multiple pathways where low abundant enzymes are able to synthesize bioactive compounds that accumulate in molar concentrations^53^. The dynamic nature of metabolons enables on-demand assembly and requires that the complexes are stabilized by low-affinity interactions. This can only be achieved by robust colocalization of the individual enzymes, as facilitated by membrane anchoring, that enables otherwise weak interactions to be stabilized and thereby evolutionary selected. We propose that the acquisition of a membrane anchor has been a nexus in the evolution of multienzyme enzyme clusters harboring several POR and P450s by facilitating evolutionary coupling of otherwise distributed and weakly interacting proteins^46^.

A similar design principle might be exploited for engineering and laboratory evolution of multienzyme pathways in heterologous systems. The synthetic biology field is rapidly developing for the production of highly complex plant-derived molecules such as cannabinoids^54–56^ and alkaloids^57^. Both of these pathways are associated with the ER membrane via P450 enzymes. However, membrane anchoring of some of the soluble enzymes could improve the coupling between sequential enzymatic steps by the concept of local concentration enhancement and render those enzymes dedicated to the production of the desired compounds. This might circumvent the challenge of some heterologous enzymes to interfere with the metabolism of the microorganism.

Notwithstanding their broad importance, we are lacking fundamental knowledge about the dynamic organization of advanced metabolic sequences (metabolons) in plants. Our platform for reconstitution and single molecule observation of transmembrane anchors opens a new front in the effort to dissect the fundamental principles governing organization of metabolic enzyme cascades on membranes. Ultimately, a more complete understanding of the role of membrane anchoring in plant metabolic circuits could enable innovative approaches to the design of synthetic biological synthesis devices.

## Methods

### Chemicals

All chemicals were of analytical grade, purchased from Sigma Aldrich (Denmark) unless stated otherwise and used as supplied. Lipids L-α-phospholcholine (Egg PC) and 1,2-dioleoyl-*sn*-glycero-3-phospho-L-serine (DOPS) were from Avanti Polar Lipids (Alabaster, AL). 1,2-dihexadecanoyl-*sn*-glycero-3-phosphoethanolamine (Texas Red® DHPE) was from Life Technologies.

### Chemical synthesis of SbPOR2B hydrophobic membrane anchor segment

The peptides were prepared by Fmoc solid-phase peptide synthesis on an automated peptide synthesizer Syro II (Biotage®). The syntheses were carried out on a TentaGel S Rink Amide 0.24 mmol/g, (Rapp Polymere GmbH) resin. The amino acids had 9-fluorenylmethyloxycarbonyl (Fmoc) for protection of Nα-amino groups. Side-chain protecting groups were *tert*-butyloxycarbonyl (Boc, for Lys and Trp) tert-butyl (OtBu for Glu), 2,2,4,6,7-pentamethyl-dihydrobenzofuran-5-sulfonyl (Pbf, for Arg), and trityl (Trt for Cys) *N*,*N*-dimethylformamide (DMF), *N*-methylpyrrolidone (NMP), *N*-[(1*H*-benzotriazol-1-yl)(dimethylamino)methylene]-*N*-methylmethanaminium hexafluorophosphate *N*-oxide (HBTU), 1-Hydroxy-7-azabenzotriazole (HOAt), piperidine and *N*,*N*-diisopropylethylamine (DIPEA). All the amino acids and reagents were supplied by Iris Biotech. Acetonitrile, formic acid (FA), triethylsilane (TES), dichloromethane (DCM), Trifluoroacetic acid (TFA), and acidic anhydride were purchased from (Sigma Aldrich). ATTO488 maleimide and ATTO647N maleimide was purchased from ATTO-TEC GmbH, Siegen. Water was purified using an Ultra Clear Water System (Siemens)

Purification was performed by RP-HPLC (Dionex Ultimate 3000 system) with preparative C4 (FeF Chemicals, 300 Å 5 µm C4 particles, 21.5 × 250 mm and C18 columns (FeF Chemicals, 300 Å 5 µm C18 particles, 21.5 × 250 mm) using the following solvent system: water containing 0.1% TFA (solvent A) and acetonitrile containing 0.1% TFA (solvent B). The purities of the peptides were evaluated on a LC–MS instrument consisting of an analytical HPLC Dionex Ultimate 3000 system) with an analytical C18 column (Phenomenex Gemini NX 110 Å, 5 µm, C18 particles, 4.60 × 50 mm) coupled to a (ESI-MS) (MSQ Plus Mass Spectrometer, Thermo), using a linear gradient flow of water- Acetonitrile containing 0.1% formic acid,) and going from five to 100% Acetonitrile over 10 min (Supplementary Fig. 2).

### Peptide synthesis

The synthesis was performed on a TentaGel S Rink Amide 0.24 mmol/g on a 0.1 mmol scale. For the Fmoc-removal piperidine-DMF (2:3) was used for 3 min, followed by 15 min with piperidine-DMF (1:3) amino acid incorporation was done using, four equivalents of amino acids and HOAt, 3.8 equivalents of coupling reagents HBTU all dissolved in DMF and 7.8 equivalents of DIEA dissolved in NMP. The coupling reaction time was 120 min and each coupling was repeated in order to achieve high purity of the crude peptide. In between couplings and deprotection, the resin was washed three times with NMP, 2× with DCM, and then 3× with NMP again.

After synthesis, the peptide was acetylated N-terminally by addition of an acidic anhydride -DMF (1:4) solution to the resin and reacting it twice for 15 min. The resin was washed with NMP (3×) and then with DCM (5×). The peptide was released from the solid support by treatment with trifluoroacetic acid (TFA), triethylsilane (ES), and H_2_O (95:2:3) for 2 h. The TFA solutions were concentrated by nitrogen flow and the compounds were precipitated with diethylether to yield the crude product. The POR2B peptide was synthesized with an N-terminal cysteine residue for maleimide conjugation chemistry of fluorescent probes.

(POR2B) Ac-CEGRALVATLAAAVLGAALFVLWRRAAAGKKRKREA-NH_2_

The crude peptide was purified on C18 HPLC column using a gradient elution (0–5 min: 5% (solvent B) to 30%; 30–60% solvent B 5–40 min) was applied at a flow rate of 15 mL min^−1^. The purified product was lyophilized.

The yield 40 mg with a purity of more than 96% assessed by LC–MS (Supplementary Fig. 2)

(POR-488) Ac-C(ATTO488)EGRALVATLAAAVLGAALFVLWRRAAAGKKRKREA-NH_2_

POR2B peptide (5 mg) was dissolved in 10 ml of 50 µM in a phosphate buffer at pH 7.0. ATTO488 (1 mg) was dissolved in 0.5 ml DMSO and added to the reaction, which was then stirred overnight at 4 °C in darkness. The subsequent mixture was purified on C4 HPLC column using gradient elution (0–5 min: 5% (solvent B) to 40%; 40–80% 5–40 min), which after lyophilization yielded 2.5 mg with a purity 96%, according to LC–MS (Supplementary Fig. 3).

(POR-647N) Ac-C(ATTO647N)-EGRALVATLAAAVLGAALFVLWRRAAAGKKRKREA-NH_2_

POR2B peptide (5 mg) was dissolved in 10 ml of 50 µM in a phosphate buffer at pH 7.0. ATTO647N (1 mg) was dissolved in 0.5 ml DMSO and added to the reaction, which was stirred overnight at 4 °C in darkness. The subsequent mixture was purified on C18 HPLC column using gradient elution (0–5 min: 5% (solvent B) to 40%; 40–80% 5–40 min) yielding after lyophilization 3 mg with a purity of 98%, according to LC–MS (Supplementary Fig. 4).

### Preparation of supported lipid bilayers

Planar supported lipid bilayers (SLBs) were formed by adsorption of small unilamellar vesicles (SUVs) on round clean glass microscope coverslips (Fischer Scientific)^58,59^. SUVs were formed from a lipid solution of 98% Egg-PC and 2% DOPS lipids in chloroform in a 10 mL round bottom glass flask. The solvent was evaporated with a rotary evaporator (Büchi) equipped with a vacuum pump for 30 min and dried under nitrogen for 30 min. Subsequently, the dried lipid film was rehydrated in deionized milli Q water to reach 1 mg/mL of lipids and vortexed for 30 min. Vesicles formed were probe-sonicated in an ice water bath three times for 45 s- and 15 s rest or until the suspension turned clear. SUVs were centrifuged at 20,000 *g* for 30 min and supernatant was recovered. Prior to deposition of SUVs, glass coverslips were rinsed^59^. Clean coverslips were etched in Piranha solution (3:1 H_2_SO_4_:H_2_O_2_) for 3 min to remove adsorbed organic material. Coverslips were placed in an open chamber and 400 μL SUVs solution (0.5 mg/mL) diluted in buffer (PBS, pH 7.5) were applied. SLB was formed spontaneously upon 30 min incubation, following wash by gentle pipetting with milli Q water to remove excess lipids.

### Preparation of giant unilamellar vesicles with POR membrane anchor peptides

Giant unilamellar vesicles (GUVs) were prepared^60^ by dissolving lipids (~98% Egg-PC, 2% DOPS, 0.005% Texas Red, and 0.0001% POR-647N) in 1:1 methanol:chloroform in a 25 mL round bottom glass flask. The solvent was evaporated as described for SUVs above with the only difference that lipids were kept dark in aluminum foil to avoid photobleaching of fluorescent labels. The dried lipid film was rehydrated by gently adding 5 mL degassed 100 mM sucrose solution. The flask was purged with nitrogen and sealed and incubated overnight at 37 °C in a water bath to allow the formation of GUVs. It is critical to avoid shaking since GUVs are very fragile and easily brakes into smaller liposomes. The GUVs were collected by pipetting of cloudy material in the 5 mL solution using a P1000 pipette tip avoiding excessive friction. Deposition of GUVs on SLBs was performed in the water allowing GUVs containing 100 mM sucrose to sediment due to higher density. 0.5 μL of recovered GUVs (unknown concentration) was added and ready for imaging within 5 min.

### Microscopy

Imaging of POR-647N peptides was achieved by total internal reflection fluorescence (TIRF) microscopy on an inverted microscope (Nikon Eclipse Ti; Technical Instruments, Burlingame, CA) with a custom-built laser launch 488 nm (Sapphire HP; Coherent Inc., Santa Clara, CA) and 640 nm (Cube; Coherent Inc.) diode laser. With a dichroic beamsplitter (z488/647 rpc; Chroma Technology Corp., Bellows Falls, VT) lasers were reflected through the objective lens (Nikon 1.49 NA TIRF; Technical Instruments, Burlingame, CA) and fluorescence images were recorded on an EM-CCD (iXon 597DU; Andor Inc., South Windsor, CT) camera^61,62^ using MetaMorph image acquisition software (Molecular Devices Inc., Downingtown, PA). Data were acquired with a pixel size of 158 nm X 158 nm per pixel with a laser power of 15 mW and exposure time 10 ms yielding an acquisition time for each frame of 16 ms. For experiments with non-fluorescent SLBs, the bilayer was imaged prior to GUV deposition and revealed little or no fluorescent background. Upon deposition, adsorbing GUVs with conical shapes giving rise to planar surfaces were selected for imaging. Typically, each position was photobleached with the 488 nm laser for 10 min with 25 mW laser power to enable single-particle tracking resolution, and frames were recorded for 100 s (6250 frames) at each position.

Confocal microscopy was performed on a custom-built spinning-disk confocal system^63^. Images were captured with a Nikon Apo TIRF 100. oil-immersion objective (1.49 NA) and an EMCCD (Andor iXon3 888), and the microscope was controlled with μManager61. The axial slice step size was 0.5 μm and pixel size of 72 nm X 72 nm per pixel.

### Single molecule tracking

Particle tracking was carried out using a software suite^63^ written in Igor Pro ver. 6.22 A. Single molecule intensity speckles were first located in each image by thresholding the divergence of the gradient vector field calculated from a smoothed (3 × 3 Gaussian filter) version of the original image. Statistical analysis on the original image was used to confirm the presence of genuine particles at each identified position of interest and, upon passing the statistical test, each speckle was fitted with a two-dimensional Gaussian function to extract x, y coordinates, and other characteristic parameters. In this way, each image in a time series was converted to a list of particle coordinates. Particles were linked between adjacent frames to create trajectories using the nearest neighbor algorithm with a maximum allowed traveled distance of 6 pixels (0.948 µm). A minimum trajectory length of 5 frames (POR-647N data) or three frames (POR-488 data) was imposed.

Step size data were fitted using a maximum likelihood modality^28^. This was achieved by calculating the joint probability of the dataset comprising step length and timestamp pairs in a free Brownian diffusion model with either one or two distinct components. The best model was selected based on the Bayesian Information Criterion (BIC) (see also ref. ^29^).

Brightness trajectories as shown in Fig. 3 were obtained by integrating the intensity in a 5×5 pixel region of interest tracing each trajectory. Traces were checked automatically for step photobleaching by change point analysis^28^. A further layer of manual curation was employed to check that traces exploring multiple brightness plateaus indeed reflected particles coming together and dissociating spatially as shown in Fig. 3b before these were selected for further analysis. All brightness traces were joined into one long trace and fitted with a two-state model utilizing a change point algorithm to segment traces into colocalized and distributed segments and thereby quantify on- and waiting-times^32^. With reference to the algorithm covered in ref. ^32^ no filtering was applied, a Bayes factor of 3 was used for level detection by the Gaussian approach and a Bayes factor of 10 was applied for the refinement^32^. The threshold for the two-state level segmentation was fixed to 1000 intensity units across the data series^32^. The same analysis procedure was used for the quantification of stochastic simulation data of colocalization and diffusion reaction.

### Stochastic simulations

Coincident colocalization was evaluated by simulating single molecule tracking time series using the same number of particles and intensity characteristics (width and amplitude of the intensity speckle) as determined for the microscopy data. Particles were initially randomly distributed over the field of view (matched to the size of the original images) and then allowed to diffuse by random sampling of the probability density for free Brownian motion in two dimensions (equation S1) using a random number generator (enoise function in Igor Pro 6.22 A). Each time lapse was 16 ms as for the original data and the simulation was continued to produce movies of the same length as the original data. The same basic approach was used for the reaction diffusion system simulations where two point particles were allowed to move randomly in two or three dimensions inside a cubic box with side length *L* = 0.25 µm. For these simulations, time increments were 0.01 ms and each simulation was 750 ms. Two molecules were taken as in contact when the center-to-center distance was ≤20 nm (see Supplementary Table 3 for further details).

### Bioinformatics

Functionally characterized and predicted full-length POR sequences from land plants (Supplementary Data 1 and 2) were retrieved from Phytozomev12^64^, One Thousand Plant Transcriptomes^65^, the National Center for Biotechnological Information (NCBI), and Uniprot. Accession numbers and database sources are given in Supplementary Data 2. A few partial sequences were wrongly annotated in some plant genomes and were manually curated to retrieve the full-length sequence. Any changes are indicated in Supplementary Data 1. Plant species were selected to cover a broad diversity of land plant phylogeny and aimed to include all full-length POR sequences from the given species.

### Statistics and reproducibility

Statistical analysis was performed using Igor Pro ver. 6.22 A. GUV reconstitution of POR anchors and single molecule tracking was reproduced in experiments conducted on three separate days and with two different fluorphore labels on the anchors. The number of GUVs and step length, time stamp pairs included in the analysis for each condition is given in the legend of the figure and/or table.

### Reporting summary

Further information on research design is available in the Nature Research Reporting Summary linked to this article.

## Supplementary information

Supplementary Information

Description of Supplementary Files

Supplementary Data 1

Supplementary Data 2

Supplementary Data 3

Supplementary Data 4

Supplementary Data 5

Supplementary Data 6

Supplementary Data 7

Reporting Summary

## Data Availability

All data is available in the main text or the supplementary materials and supplemetary data. Raw data in the format of.csv or.tif files are available from the corresponding author upon reasonable request.

## Code availability

Code used in this work has been previously published as referenced in the Methods. The procedure for stochastic simulations is described and can be implemented in a given coding language.
