# Supplementary material for: Membrane anchoring facilitates colocalization of enzymes in plant cytochrome P450 redox systems
Source: Commun Biol. 2021 Sep 9;4:1057. doi: 10.1038/s42003-021-02604-1 (PMC8429664; doi:10.1038/s42003-021-02604-1)
Supplement: Supplementary file 1 — Supplementary Information [file 42003_2021_2604_MOESM1_ESM.pdf]

Membrane anchoring facilitates colocalization of enzymes in plant cytochrome P450 redox systems

Supplementary Information

Tomas Laursen[1], Hiu Yue Monatrice Lam[2], Kasper Kildegaard Sørensen[3], Pengfei Tian[4], Cecilie Cetti Hansen[1], Jay T. Groves[2], Knud Jørgen Jensen[3] and Sune M. Christensen[2,4*]

[1]Department of Plant and Environmental Sciences, University of Copenhagen, Denmark

[2]Department of Chemistry, University of California, Berkeley, USA

[3]Department of Chemistry, University of Copenhagen, Denmark

[4]Present address: Enzyme Research, Novozymes A/S, 2800 Kgs. Lyngby, Denmark

[*]Correspondence to scr@novozymes.com

Supplementary Note 1: Physiological POR membrane density

Characteristic surface density of protein in cellular membranes is in the range 30,000-40,000 proteins μm^{-2} [Supplementary References 1,2]. Quantitative proteomics data [Supplementary References 3] show that POR comprise 0.18±0.02% of the microsomal protein yielding an estimated POR density in *Sorghum bicolor* ER membranes of ca. 45-80 μm^{-2}. Correspondingly, the total P450 density (1.2% of microsomal membrane [Supplementary References 3]) is estimated to be ca. 300-550 μm^{-2}.

Supplementary Note 2: Diffusion analysis

For the diffusion analysis we pooled step length vs timestamp pairs (r,t) for each condition. We considered either a single-component free Brownian diffusion model (equation S1) or a two-component free Brownian diffusion model (equation S2). In both cases we calculated the joint probability of observing the data, given the model, based on the probability densities. The joint probability was evaluated on a grid with different values of the model parameters (diffusion coefficients and, in the case of the two-component model, alpha (α), which described the fraction of the data characterized by diffusion coefficient D1). In all cases, the best estimate of the model parameters was obtained as the value that optimized the joint probability in accordance with the principle of maximum likelihood. In the single-component free Brownian model, the joint probability was evaluated as a function of the parameter D on a grid spanning 0-5 um2/s with 0.05 increments. In the two-component free Brownian model, the joint probability was evaluated as a function of the parameters D1and D2 on a grid spanning D=0-5 um2/s with 0.05 increments, whereas alpha was evaluated on a grid spanning 0-0.5 with 0.01 increments. The procedure has been described by Lin *et al.*[Supplementary References 4]. As described in the previous publication, we used Baye's rule to iteratively update the posterior probability of observing the data by looping through chunks of (r,t) pairs and using the posterior probability from each iteration as the prior for the next iteration. The first iteration was carried out using a flat prior.

$$\rho(r,t,D)=\frac{r}{2Dt}\exp\left(-\frac{r^2}{4Dt}\right)$$

Equation S1

$$\rho(r,t,D_1,D_2,\alpha) = \frac{\alpha r}{2D_1 t}\exp\left(-\frac{r^2}{4D_1 t}\right) + \frac{(1-\alpha)r}{2D_2 t}\exp\left(-\frac{r^2}{4D_2 t}\right)$$ Equation S2

We used the Bayesian Information Criterion (BIC) to decide whether the most appropriate model for the data was one- or two-component Brownian motion, equation S3 (with *k* indicating the number of model parameters, n the sample size and L the maximized value of the likelihood function, see also Bohr *et al*. [Supplementary References 5]. The model with the lower BIC is favored. A ΔBIC >10 between two models is considered “very strong” evidence in favor of the model with the lower BIC in that comparison. As can be seen in Supplementary Table 1, we have very strong evidence for two-component free Brownian motion as being the most proper model describing the anchor diffusion data. As expected, the single component free Brownian motion simulated data produced very strong evidence for the single-component model.

$$\mathrm{BIC} = k\ln(n) - 2\ln(L)$$ Equation S3

Supplementary Note 3: Colocalization enhancement at the membrane

Colocalization of two particles of radius R in a cubic box with length L in 2- or 3 dimensions is given by:

$$P^{2D}_{coloc} = \frac{\pi(2R)^2}{L^2}$$

$$P^{3D}_{coloc} = \frac{4\pi(2R)^3}{3L^3}$$

The enhancement in colocalization upon confinement to a plane (the bottom of the cube) is then simply the ratio of the two:

$$FoldX = \frac{P^{2D}_{coloc}}{P^{3D}_{coloc}} = \frac{3L}{8R}$$ Equation S4

$$C_{bulk} = \frac{2}{L^3}$$

$$FoldX(C) = \frac{3}{2^{8/3}R\sqrt[3]{C_{bulk}}}$$ Equation S5

Sample	Number of data points in analysis (StepLength, TimeStamp)	ΔBIC ($BIC_{1-component}$ - $BIC_{2-component}$)	Preferred model (1- or 2-component free Brownian)	D1 ($\mu m^2/s$)	D1 HDI95 ($\mu m^2/s$)	D2 ($\mu m^2/s$)	D2 HDI95 ($\mu m^2/s$)	Alpha	Alpha HDI95
POR-647N	11444	522	2	0.55	0.50-0.59	2.60	2.55-2.65	0.24	0.23-0.26
POR-488	2960	108	2	0.10	0.05-0.15	1.15	1.10, 1.23	0.11	0.09-0.13
Simulated data (single Brownian)	8727	-18	1	2.35	2.25-2.45				

Supplementary Table 1 – Tabulated data pertaining to the mobility analysis. For Atto647N and Atto488 tracking data were pooled from movies acquired at, respectively, 3 (POR-647N) or 4 (POR-488) different GUVs. Delta BIC indicates the difference in the Bayesian Information Criterion calculated for, respectively, a one- or two-component free Brownian diffusion model fit of the data. HDI95 indicates the 95% Highest Density Interval (error bar) obtained from the posterior probability density used to estimate the parameter values by the maximum likelihood principle.

**Sample** Anchor-Atto647N	**Number of data points in analysis (StepLength,TimeStamp)**	**D1 ($\mu m^2/s$)**	**D1 HDI95 ($\mu m^2/s$)**	**D2 ($\mu m^2/s$)**	**D2 HDI95 ($\mu m^2/s$)**	**Alpha**	**Alpha HDI95**
Colocalized	149	1.25	0.90-1.75	3.30	2.50-4.50	0.62	0.42-0.78
Distributed	3455	0.80	0.66-0.95	2.65	2.50-2.80	0.29	0.25-0.33

Supplementary Table 2 – Tabulated data pertaining to the mobility analysis for a data set split according to colocalized and distributed segments based on the brightness analysis. The colocalized data has a higher proportion of data pertaining to the slow mobility species (alpha=62% in D1 distribution as compared to alpha=29% for distributed data).

Geometry	D (um2/s)	Nevents	Nruns	Collision frequency (s-1)	Average lifetime (ms)	Colocalization %
2D	0.02	145	3	64 +/- 45	19 +/- 11	1.54
2D	0.1	329	3	146 +/- 65	12 +/- 3	1.6
2D	0.2	976	5	260 +/- 114	8 +/- 1	2.13
3D	1	138	3	61 +/- 16	2.6 +/- 0.5	0.16
3D	2	181	3	80 +/- 17	2.1 +/- 0.5	0.15
3D	7	334	3	148 +/- 30	1.23 +/- 0.01	0.18

Supplementary Table 3 – Tabulated data for the simulation of diffusion in 2D or 3D. All simulations were done with a cubic box with side L=0.25 μm and 2 molecules in the box corresponding to a concentration of 206 nM and a membrane density of 31 molecules/μm^2. Time increments in the stochastic simulation were 0.01 ms and each simulation run was 750 ms. The two molecules were counted as colocalized when the center-to-center distance was ≤20 nm.

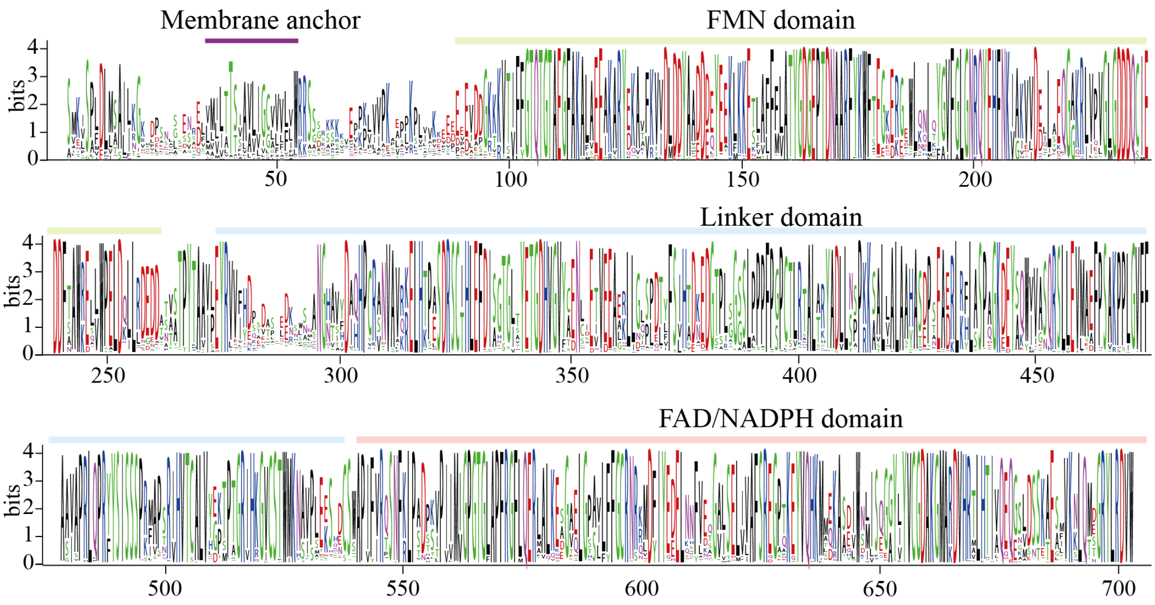

>POR
MDSATTSGAMELVAALLRGRVPPELMGGDGAEGR***ALVATLAAAVLGAALFVLWR***RAAAGKKRKREAAAAAVAEATEVKARAAKGGEDEKAADDGRKKVTV 100
FFGTQTGTAEGFAKALAEEAKARYDKAIFKVVDLDDYAAEDEEYEEKLKKEKLALFFVATYGDGEPTDNAARFYKWFTEGNERGVWLNDFEYAVFGLGNR 200
QYEHFNKVAKVVDEILTEQGGKRLVPVGLGDDDQCIEDDFNAWKEALWPELDRLLRDENDASTGTTYTAAIPEYRVEFIKPEEAAHLERNFSLANGHAVH 300
DAQHPCQANVAVRRELHTPASDRSCTHLEFDIAGTGLTYETGDHVGVYTENCPEVVEEAERLLGYSPDTFFTIHADKEDGTPLSGSSLAPPFPSPITVRN 400
ALARYADLLNSPKKTSLVALATYASDPAEADRLRFLASAAGKDEYAQWVVASQRSLLEVMAEFPSAKPPLGVFFAAVAPRLQPRYYSISSSPSMAATRIH 500
VTCALVHETTPAGRVHKGVCSTWIKNAVPSEESKDCSWAPIFVRQSNFKLPADPSVPIIMIGPGTGLAPFRGFLQERLAQKESGAELGPSVFFFGCRNSK 600
MDFIYEDELNNFLEQGALSELVLAFSRQGPTKEYVQHKMAQKASEIWDMISQGAYIYVCGDAKGMARDVHRVLHTIVQEQGSLDSSKAESFVKNLQMEGR 700
YLRDVW

Supplementary Figure 1. WebLogo representation POR sequences with domain annotation and the amino acid sequence of Sorghum bicolor POR2B is shown below. The weblogo is based on alignment of 278 full-length land plant POR sequences.

POR2B

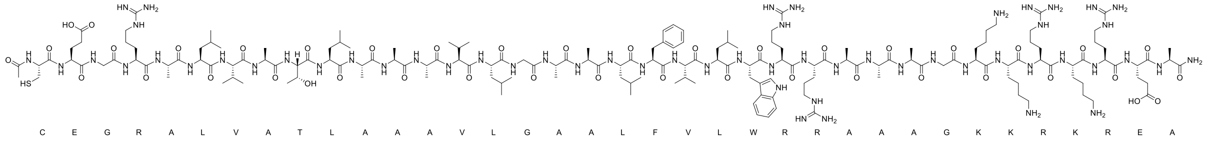

Calculated for Chemical Formula $C_{171}H_{292}N_{56}O_{42}S$, Molecular Weight: 3836,6 Da; ESI-MS, found masses: m/z: 1278.1 $[M+3H]^{4+}$, 958.9 $[M+4H]^{4+}$, 767.2 $[M+5H]^{5+}$

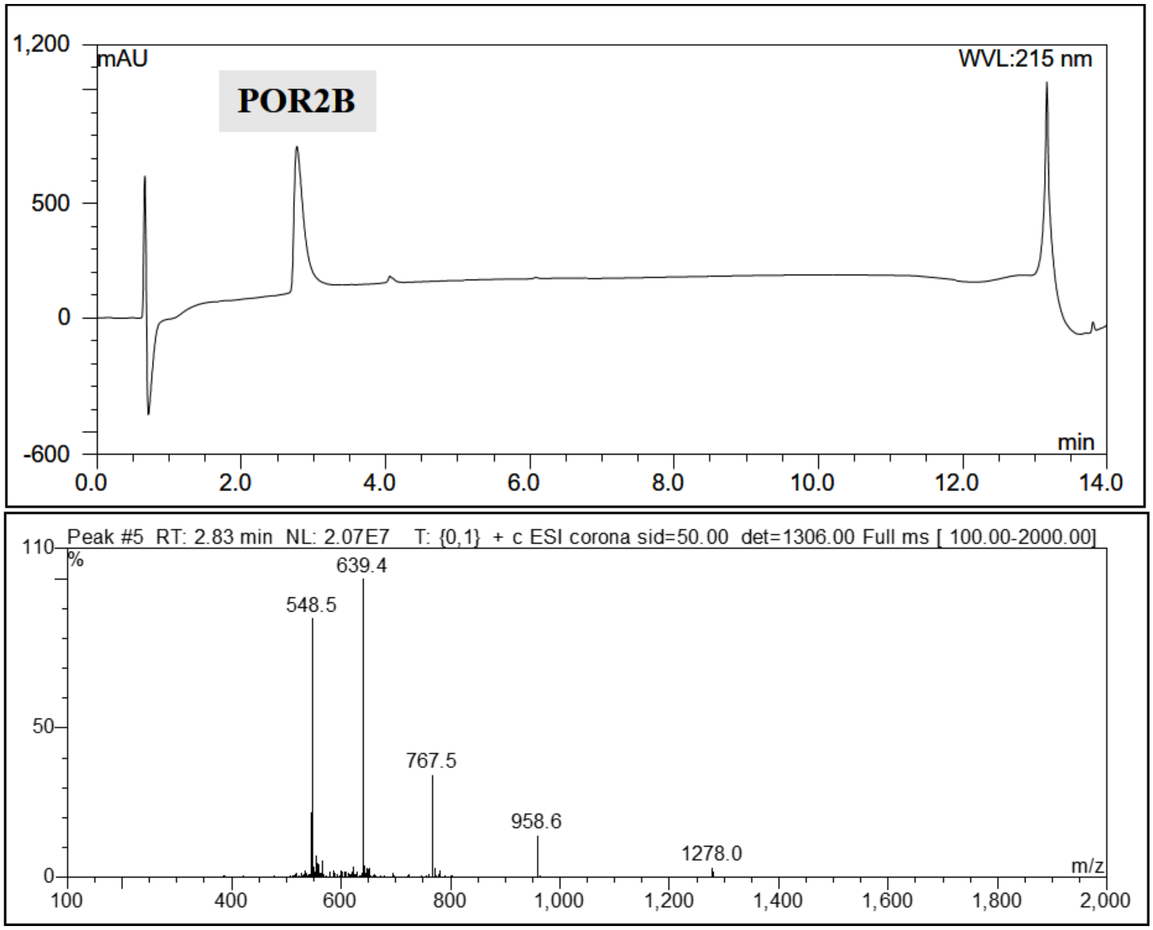

Supplementary Figure 2. Characterization of synthetic POR2B membrane anchors by LC-MS. Chemical synthesis of the 36 amino acid peptide. The amino acid sequence is illustrated with the corresponding LC-MS chromatogram.

POR-488

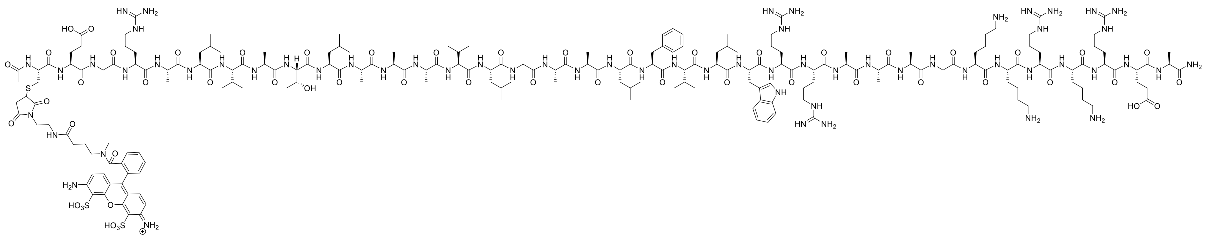

Calculated for Chemical Formula: $C_{202}H_{322}N_{61}O_{53}S_3^{+}$, Molecular Weight: 4549.3Da; ESI-MS, found masses: m/z: 1515.8 $[M+3H]^{3+}$, 1136.8 $[M+4H]^{4+}$, 909.8 $[M+5H]^{5+}$

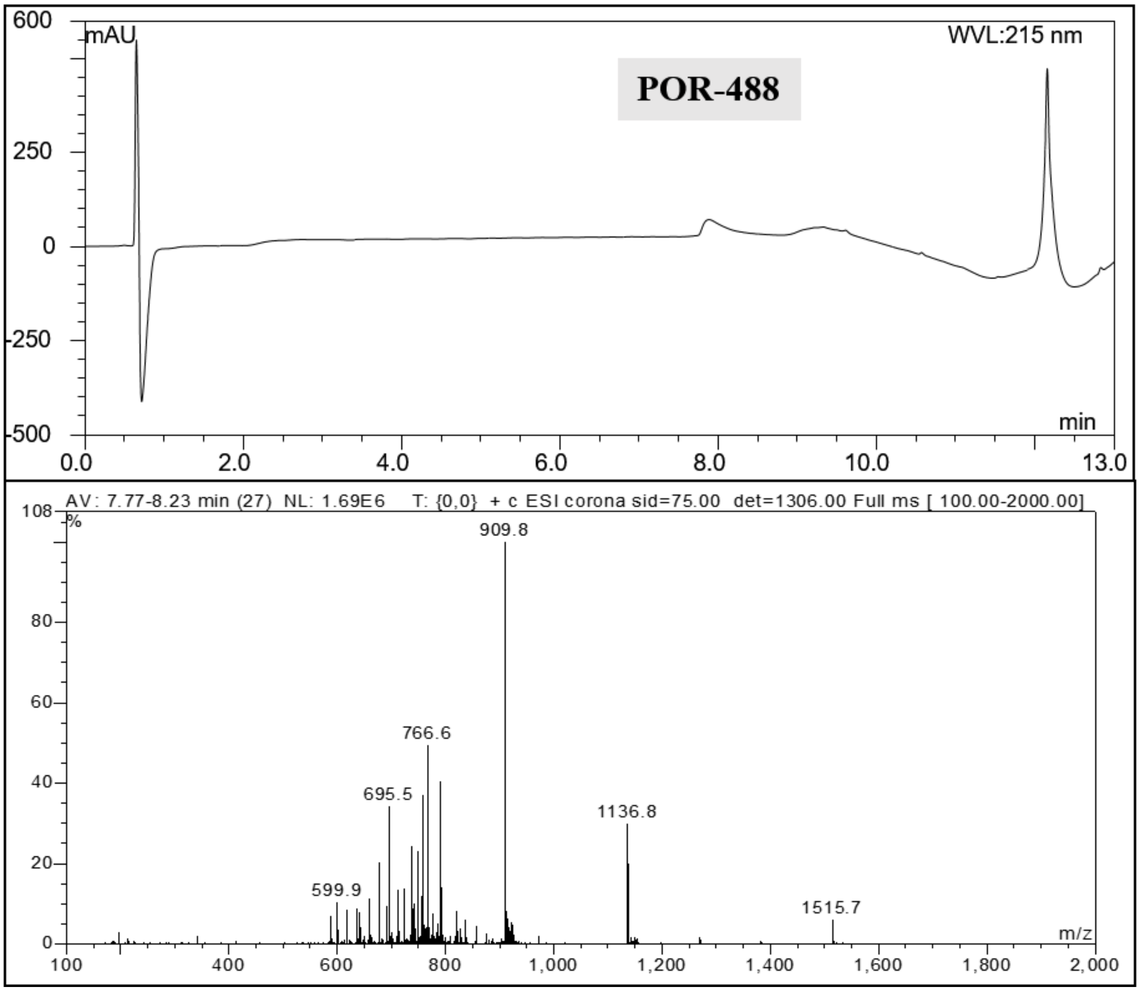

Supplementary Figure 3. Characterization of synthetic POR-488 membrane anchors by LC-MS. Chemical synthesis of the 36 amino acid peptide with N terminal modification with ATTO488. The amino acid sequence is illustrated with the corresponding LC-MS chromatogram.

POR-647N

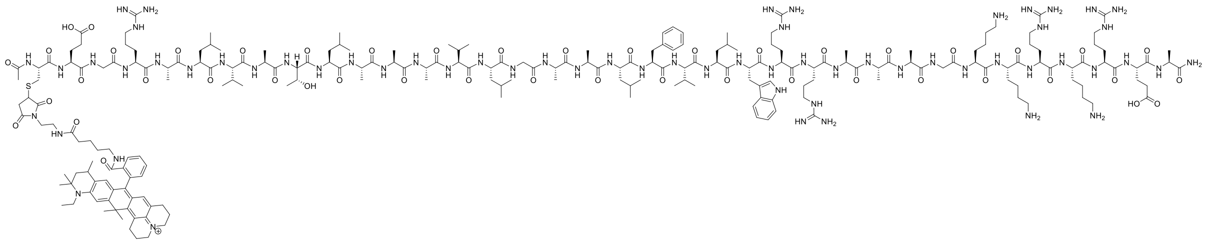

Calculated for Chemical Formula: $C_{219}H_{350}N_{61}O_{46}S^{+}$, Molecular Weight: 4605.6 Da; ESI-MS, found masses: m/z: 1151.0 $[M+4H]^{4+}$, 921.0 $[M+5H]^{5+}$, 767.3 $[M+6H]^{6+}$, 658.4 $[M+7H]^{7+}$, 575.7 $[M+8H]^{8+}$

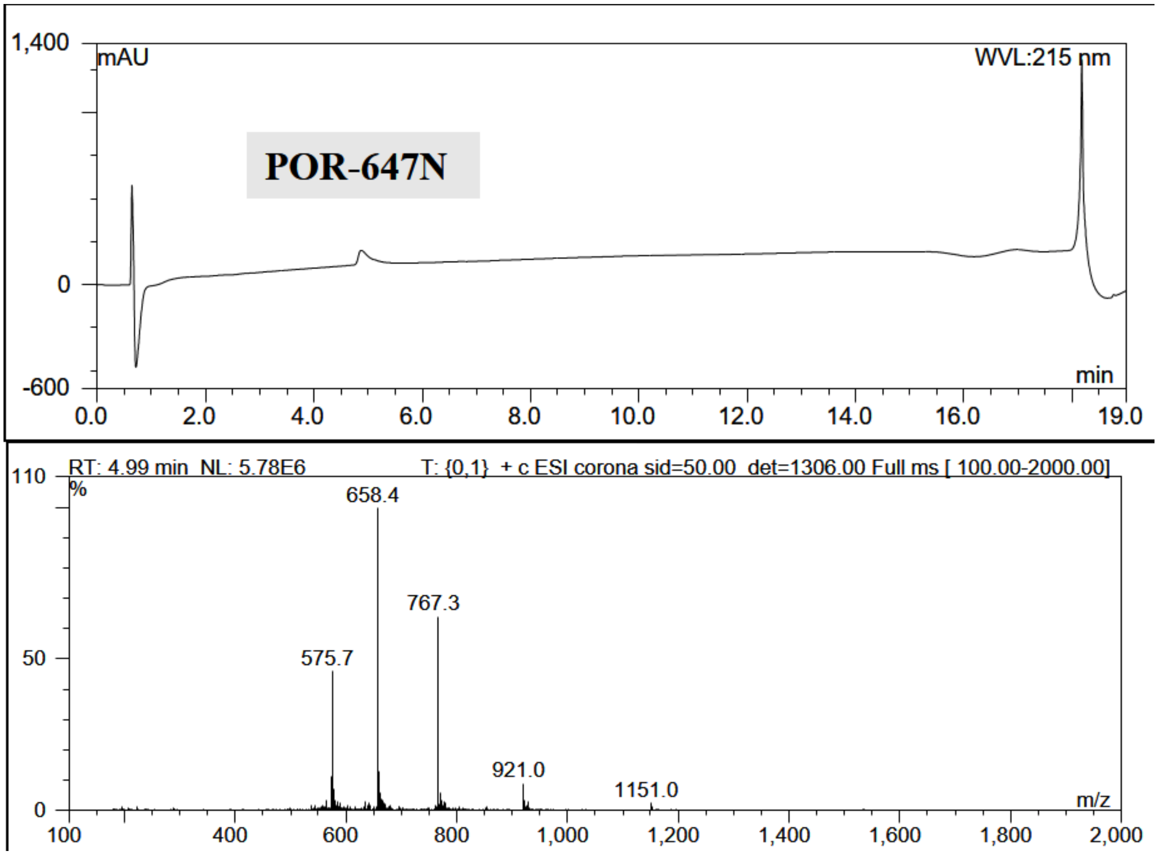

Supplementary Figure 4. Characterization of synthetic POR-647N membrane anchors by LC-MS. Chemical synthesis of the 36 amino acid peptide with N terminal modification ATTO647N. The amino acid sequence is illustrated with the corresponding LC-MS chromatogram.

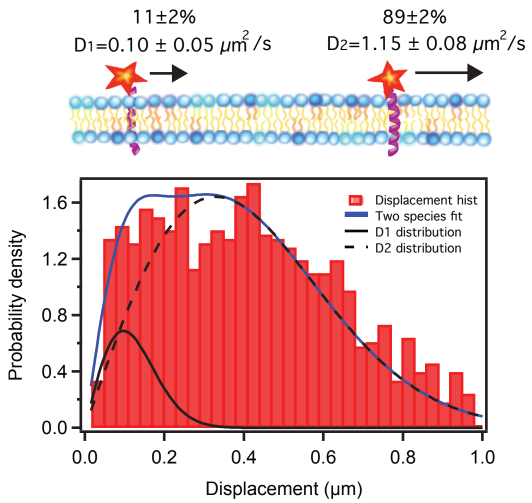

Supplementary Figure 5. Displacement histogram for single molecule tracking of POR-488 anchors. Histogram shows displacements for t=47 ms time lapse. See Supplementary Table 1 for sample size and fit information.

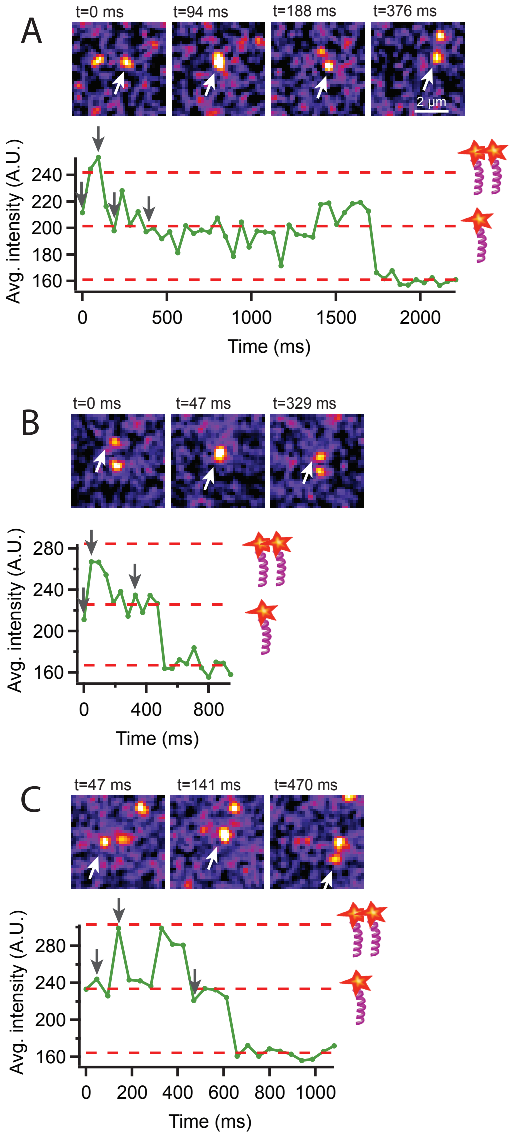

Supplementary Figure 6. Dynamic colocalization of POR-488 anchors. White arrows on the micrographs mark the tracked particle with the corresponding time stamps as indicated by the grey arrows on the intensity traces.

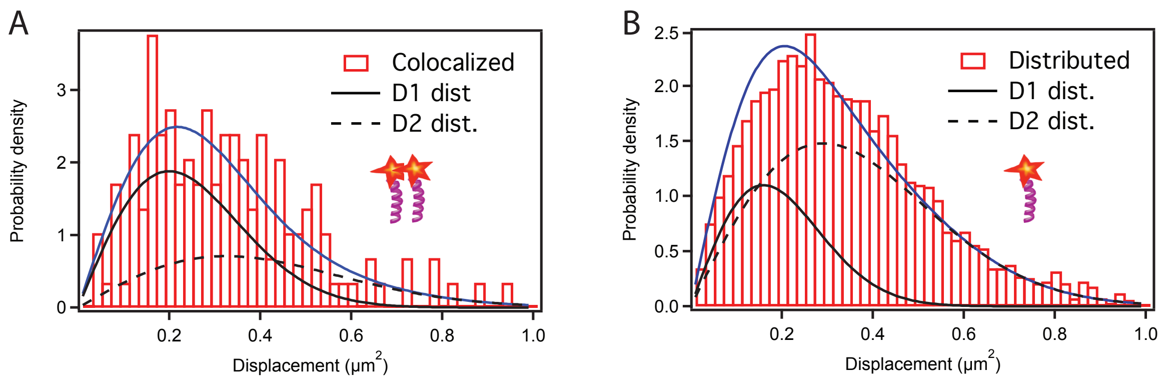

Supplementary Figure 7. Displacement histograms for data split based on the brightness-based trace segmentation into colocalized and distributed regions, c.f. main figure 3b. The shown fits depict the slow and the fast diffusing species, according to the fit parameters given in Supplementary Table 2.

Supplementary References

1 Bassard, J. E., Moller, B. L. & Laursen, T. Assembly of Dynamic P450-Mediated Metabolons-Order Versus Chaos. Curr Mol Biol Rep 3, 37-51, (2017).

2 Guigas, G. & Weiss, M. Effects of protein crowding on membrane systems. Biochim Biophys Acta 1858, 2441-2450, (2016).

3 Laursen, T. *et al.* Characterization of a dynamic metabolon producing the defense compound dhurrin in sorghum. Science 354, 890-893, (2016).

4 Lin, W. C. *et al.* H-Ras forms dimers on membrane surfaces via a protein-protein interface. Proc Natl Acad Sci U S A 111, 2996-3001, (2014).

5 Bohr, S. S. *et al.* Direct observation of Thermomyces lanuginosus lipase diffusional states by Single Particle Tracking and their remodeling by mutations and inhibition. Scientific reports 9, 16169, (2019).
