# Supplementary material for: Membrane anchoring facilitates colocalization of enzymes in plant cytochrome P450 redox systems
Source: Commun Biol. 2021 Sep 9;4:1057. doi: 10.1038/s42003-021-02604-1 (PMC8429664; doi:10.1038/s42003-021-02604-1)
Supplement: Supplementary file 2 — Description of Supplementary Files [file 42003_2021_2604_MOESM2_ESM.pdf]

Description of Additional Supplementary Files

File name: Supplementary Data 1
Description: *Amino acid sequences of POR enzymes from diverse plant species.* Each sequence is listed along with plant species name and gene ID/accession number. Further sequence information is provided in Supplementary Data 2.

File name: Supplementary Data 2

Description: *List of plant species that were included in the study of conservation of plant POR membrane anchors.* Accession numbers/gene IDs are provided for each of the sequences included in the analysis. The amino acid sequences are listed in Supplementary Data 1. DOI links to scientific articles are provided for POR enzymes that have been functionally characterized.

File name: Supplementary data 3

Description: Main figure 1c data

File name: Supplementary data 4

Description: Main figure 1d data_ Sequence alignment input for generating WebLogo

File name: Supplementary data 5

Description: Main Figure 2 data

File name: Supplementary data 6

Description: Main Figure 3 data

File name: Supplementary data 7

Description: Main Figure 4 data
