# Supplementary material for: Membrane anchoring facilitates colocalization of enzymes in plant cytochrome P450 redox systems
Source: Commun Biol. 2021 Sep 9;4:1057. doi: 10.1038/s42003-021-02604-1 (PMC8429664; doi:10.1038/s42003-021-02604-1)
Supplement: Supplementary file 10 — Reporting Summary [file 42003_2021_2604_MOESM10_ESM.pdf]

nature research

Corresponding author(s): Sune M. Christensen

Last updated by author(s): Jul 1, 2021

Reporting Summary

Nature Research wishes to improve the reproducibility of the work that we publish. This form provides structure for consistency and transparency in reporting. For further information on Nature Research policies, see our Editorial Policies and the Editorial Policy Checklist.

Statistics

For all statistical analyses, confirm that the following items are present in the figure legend, table legend, main text, or Methods section.

n/a	Confirmed	
☐	☒	The exact sample size (*n*) for each experimental group/condition, given as a discrete number and unit of measurement
☐	☒	A statement on whether measurements were taken from distinct samples or whether the same sample was measured repeatedly
☒	☐	The statistical test(s) used AND whether they are one- or two-sided<br>*Only common tests should be described solely by name; describe more complex techniques in the Methods section.*
☒	☐	A description of all covariates tested
☒	☐	A description of any assumptions or corrections, such as tests of normality and adjustment for multiple comparisons
☐	☒	A full description of the statistical parameters including central tendency (e.g. means) or other basic estimates (e.g. regression coefficient) AND variation (e.g. standard deviation) or associated estimates of uncertainty (e.g. confidence intervals)
☒	☐	For null hypothesis testing, the test statistic (e.g. *F*, *t*, *r*) with confidence intervals, effect sizes, degrees of freedom and *P* value noted<br>*Give P values as exact values whenever suitable.*
☐	☒	For Bayesian analysis, information on the choice of priors and Markov chain Monte Carlo settings
☒	☐	For hierarchical and complex designs, identification of the appropriate level for tests and full reporting of outcomes
☒	☐	Estimates of effect sizes (e.g. Cohen's *d*, Pearson's *r*), indicating how they were calculated

Our web collection on statistics for biologists contains articles on many of the points above.

Software and code

Policy information about availability of computer code

Data collection	Microscopes were operated by either µManager61 or MetaMorph 7.5 (Molecular Devices Inc., Downingtown, PA).
Data analysis	Data were analyzed in Igor Pro ver. 6.22A using previously developed procedures as follows. Single molecule tracking was conducted as described in (Christensen et al., Nat. Struct. Mol. Biol, 2016, doi:10.1038/nsmb.3275). Diffusion analysis was conducted as described in (Lin et al., PNAS, 2014, doi/10.1073/pnas.1321155111). Segmentation of brightness traces was conducted as described in (Christensen et al., Nano Letters, 2016, DOI: 10.1021/acs.nanolett.6b00969). The simple procedure for stochastic simulations is described in the present work and can be implemented in a given coding language.

For manuscripts utilizing custom algorithms or software that are central to the research but not yet described in published literature, software must be made available to editors and reviewers. We strongly encourage code deposition in a community repository (e.g. GitHub). See the Nature Research guidelines for submitting code & software for further information.

Data

Policy information about availability of data

All manuscripts must include a data availability statement. This statement should provide the following information, where applicable:

- Accession codes, unique identifiers, or web links for publicly available datasets
- A list of figures that have associated raw data
- A description of any restrictions on data availability

All data is available in the main text or the supplementary materials. Raw data in the format of .csv or .tif files are available upon request.

Field-specific reporting

Please select the one below that is the best fit for your research. If you are not sure, read the appropriate sections before making your selection.

- [x] Life sciences
- [ ] Behavioural & social sciences
- [ ] Ecological, evolutionary & environmental sciences

For a reference copy of the document with all sections, see nature.com/documents/nr-reporting-summary-flat.pdf

Life sciences study design

All studies must disclose on these points even when the disclosure is negative.

Sample size	Sample size for the mobility analysis is listed in Figure 2 legend and Table S1. Tracking data are derived from three movies acquired from different vesicles.No statistical methods were applied to predetermine the sample size, however, acquisition of tracking data from three independent vesicles yielded a sufficient number of tracks to perform robust data analysis. Colocalization analysis: Sample size for the colocalization is listed in Figure 3 legend and Table S3.
Data exclusions	No data were pre excluded in the analysis. Particle tracking data were selected based on an automated software suite written in Igor Pro ver. 6.22A as previously described (Lin et al., PNAS, 2014, doi/10.1073/pnas.1321155111;Christensen et al., Nat. Struct. Mol. Biol, 2016, doi:10.1038/nsmb.3275). Particles were linked between adjacent frames to create trajectories using a nearest neighbor algorithm with a maximum allowed traveled distance of 6 pixels (0.948 µm). A minimum trajectory length of 5 frames (ATTO647N data) or 3 frames (ATTO488 data) was imposed. Traces were checked automatically for step photobleaching by change point analysis (Lin et al. 2014). A further layer of manual curation was employed to check that traces exploring multiple brightness plateaus indeed reflected particles coming together and dissociating spatially as shown in Figure 3B before these were selected for further analysis.
Replication	All tracking experiments were conducted multiple times and derived from independent preparations of GUVs. The results are therefore considered reproducible.
Randomization	Randomization of samples is not relevant to this study as we are specifically studying the diffusion behavior of a single sample type namely a fluorescently labeled peptide.
Blinding	Blinding of samples is not relevant to this study as we are specifically studying the diffusion behavior of a single sample type namely a fluorescently labeled peptide.

Reporting for specific materials, systems and methods

We require information from authors about some types of materials, experimental systems and methods used in many studies. Here, indicate whether each material, system or method listed is relevant to your study. If you are not sure if a list item applies to your research, read the appropriate section before selecting a response.

Materials & experimental systems

n/a	Involved in the study
[x]	[ ] Antibodies
[x]	[ ] Eukaryotic cell lines
[x]	[ ] Palaeontology and archaeology
[x]	[ ] Animals and other organisms
[x]	[ ] Human research participants
[x]	[ ] Clinical data
[x]	[ ] Dual use research of concern

Methods

n/a	Involved in the study
[x]	[ ] ChIP-seq
[x]	[ ] Flow cytometry
[x]	[ ] MRI-based neuroimaging
